# Lower limb cutaneous melanoma surgery: location matters

**DOI:** 10.1007/s00403-023-02571-z

**Published:** 2023-03-02

**Authors:** Antonio Piñero-Madrona, Pablo Cerezuela-Fuentes, Guadalupe Ruiz-Merino, Enrique Martínez-Barba, Sebastián Ortiz-Reina, María José Martínez-Ortiz, Angel López-Ávila, José F. Frías-Iniesta, Alice Viney, Juan Cabezas-Herrera

**Affiliations:** 1 Surgery Department, Hospital Clínico Universitario “Virgen de la Arrixaca”, School of Medicine, University of Murcia, Campus de Ciencias de la Salud. Edificio Departamental, Office 4.27, 30120 Murcia, Spain; 2 Instituto Murciano de Investigación Biosanitaria (IMIB), Murcia, Spain; 3 Medical Oncology Department, Hospital Clínico Universitario “Virgen de la Arrixaca”, Murcia, Spain; 4 Biostatistics Department, Fundación para la Formación e Investigación Sanitaria de la Región de Murcia, Murcia, Spain; 5 Pathology Department, Hospital Clínico Universitario “Virgen de la Arrixaca”, Murcia, Spain; 6 Pathology Department, Complejo Hospitalario de Cartagena, Cartagena, Spain; 7 Medical Oncology Department, Complejo Hospitalario de Cartagena, Cartagena, Spain; 8 Dermatology Department, Complejo Hospitalario de Cartagena, Cartagena, Spain; 9 Dermatology Department, Hospital Clínico Universitario “Virgen de la Arrixaca”, Murcia, Spain; 10 Pharmacy Department, Complejo Hospitalario de Cartagena, Cartagena, Spain; 11 Research Department, Instituto Murciano de Investigación Biosanitaria (IMIB), Murcia, Spain

**Keywords:** Cutaneous melanoma, Lower limb, Foot, Prognostic

## Abstract

The anatomical location of cutaneous melanoma is a relevant independent prognostic factor in melanoma. The aim of the study is to know the prognosis of lower limb cutaneous melanoma related to their location within the limb, regardless of the histological type, and if there are any other influencing variables. A real-world data observational study was developed. The lesions were divided depending on the location of the melanoma (thigh, leg and foot). Bivariate and multivariate analysis were performed, and melanoma-specific survival and disease-free survival rates were calculated. When these analysis were done, the results showed that, in melanomas of the lower limb, location on the foot presented a lower melanoma-specific survival rate compared to more proximal locations, and only the anatomical location presents statistical significance to discriminate cases with a higher mortality risk and a lower disease-free survival rate among distal melanomas (mainly on the foot). In conclusion, this study confirms that a more distal location of lower limb cutaneous melanoma is a relevant prognostic factor.

Trial registration number NCT04625491 retrospectively registered.

## Introduction

Melanoma is the form of skin cancer with the worst prognosis and, in recent decades, an increase has been reported in both the incidence and mortality rates [1].

Among the different prognostic factors, the anatomical location of the primary tumor has seen to be a relevant independent factor in same stage cancer, with worse survival rates in melanoma of the head and neck or trunk than those in melanoma of the limb [2, 3]. Differences in the clinical course of melanomas in different anatomical sites have been explained due to several factors: the length of lymphatic tracts and the number of lymph nodes [4], the location in regions barely noticed during self-exploration, etc. [5, 6].

Nearly 30% of all primary skin melanomas affect the lower limbs [7]. Among melanomas of the lower extremities, those arising on the feet could be related to a poorer survival rate and some authors suggest that they should be considered separately [8, 9]. Nowadays, some authors consider it is still not clear whether melanoma on the foot should be considered a separate entity with a worse prognosis [8]. Other studies have emphasized the worse prognosis of melanoma of the plantar surface of the foot [10], although in other series, their results are controversial [6].

Taking into account all of these factors, it could be hypothesized that for melanomas of the lower limb, the more distal the location of the melanoma the worse the prognosis. Most of these studies are reports from several single institutions, with non-homogeneous series, and differences in the definition of variables, such as the definition criteria for acral melanoma. This is because acral melanoma can be defined according to two different criteria: on the one hand those melanomas that are located in the most distal regions of the limbs (palms, soles and nails), whether superior or inferior, defined as acral melanomas by some authors regardless of their histology; however, for other authors [11], the diagnosis of acral melanoma must meet specific histopathological criteria, such as the existence of a hyperplastic epidermis and atypical melanocytic cell proliferation with prominent dendrites that extend throughout the epidermis, regardless of the location of the melanoma.

This study aims to analyze the effect of anatomical location on the prognosis of cutaneous melanoma of the lower limbs.

## Materials and methods

An observational retrospective multicentre real-world study was developed in a series of consecutive patients, from 2002 to 2018, with a diagnosis of lower limb cutaneous melanoma. This study was conducted in the “Virgen de la Arrixaca” University Hospital in Murcia and the University Hospital Complex in Cartagena. Patients with lower limb cutaneous melanoma without clinical or radiological detection of lymph node involvement or metastatis were included in the study. The sentinel lymph node biopsy (SLNB) was indicated in cases with a Breslow index equal to or greater than 1 mm, or if there were risk factors for lymph node involvement in cases with an index less than 1 mm, such as the presence of ulceration or a mitotic count greater than or equal to 1 mitosis/mm^2^. The SLNB procedure was carried out simultaneously with the expansion of surgical margins, following the method described in previous publications [12]. Approval by the IRB was obtained prior to developing the study and it is registered in ClinicalTrials.gov (NCT04625491). The study has been reported in line with the STROBE criteria.

The inclusion criteria was primary melanoma located in the lower limb with patients being divided into three groups for analysis: (1) thigh or proximal group: melanoma located on the thigh, including the gluteal region and root of the thigh; (2) leg or middle group: melanoma found on the leg, including the knee and popliteal socket; and (3) foot or distal group: melanoma located on the foot, including the ankle. Patients with less than a 2-year follow-up and those with insufficient recorded data (less than 80%) were excluded.

The following variables were collected: sex and age at diagnosis, histological type (superficial spreading melanoma (SSM), nodular melanoma (NM), acral-lentiginous melanoma (ALM), and lentigo maligna melanoma (LMM)), size (the largest diameter in mm), Breslow index and Clark level, ulceration, regression, the number of mitosis/mm^2^, the presence of an inflammatory infiltrate and its intensity (mild/intense), satellitosis, lymphovascular infiltration, perineural infiltration, or both (LVPNI), the number of drainage basins found, as well as lymph node involvement after studying the sentinel node. In this case, the number of affected nodes after lymphadenectomy was collected. The 7th edition of the TNM classification was used due to the period considered for patients registration.

For the follow-up of patients, disease-free survival (DFS) was defined, that is, time from surgery until the date of first recurrence (local, regional or distant metastases), and melanoma-specific survival (MSS) until death from a melanoma-related cause, both measured in months.

Statistical methodology: a descriptive study of the different variables was carried out with proportions and percentages being used for qualitative variables and mean and dispersion measurements being used for the quantitative variables, both for the global population and for the study group. Bivariate analysis of all the variables in contrast with the group of melanoma located on the lower limb was applied to verify the homogeneity of the groups. Statistical differences for qualitative and quantitative variables were analyzed using the Chi-square test and the *t* Student test respectively. Besides this, a multivariate analysis using binary logistic regression was performed including only the significant variables in the bivariate analysis. DFS and MSS analysis performed using Kaplan–Meier curves, as well as Cox regression for the different variables, with calculations of the survival rates at 5 and 10 years and their confidence intervals. For patients whose disease progressed or who died, the duration of objective follow-up was censored at the same time. Statistical analysis was performed using the SPSS (version 12.0) and a *p* value of less than 0.05 was considered statistically significant.

## Results

A total of 221 cases were included in the study distributed into 48 (21.7%) cases of proximal cutaneous melanomas, 113 (51.1%) cases of middle location melanomas and 60 (27.1%) cases of distal melanomas. The mean follow-up, was 104.25 ± 67.34 (range 2 6–369) months. All patients were Caucasian.

Table 1 shows the general characteristics of the patients and the bivariate analysis for the different melanoma locations of the lower limb for each of the different groups that were considered. Significant differences can be found for the different histological types, with ALM being more frequent in the distal group and SSM in the middle location group (leg), compared to the other two (100% and 58.3% respectively; *p* < 0.0001). For the rest of the histopathological variables, relationships could be observed between the size and Breslow depth of the lesions, which were significantly higher for most of the distal lesions (13.98 ± 10.02 mm—*p* = 0.036—and 2.71 ± 2.13 mm—*p* < 0.0001—, respectively), and the presence of ulceration, which was also more frequently associated with distal lesions (40.0%, *p* = 0.008).

**Table 1:** General characteristics of the series and bivariate analysis for the different anatomical locations of the lower limb

	Global	Thigh, *n* = 48	Leg, *n* = 113	Foot, *n* = 60	*p*
Age	54.95 ± 16.30	50.79 ± 15.92	55.31 ± 16.26	57.62 ± 16.30	0.091
Sex					0.637
Male	160 (72.4%)	37 (23.1%)	79 (49.4%)	44 (27.5%)	
Female	61 (27.6%)	11 (18.0%)	34 (55.7%)	16 (26.2%)	
Laterality					0.674
Right	106 (48.0%)	25 (23.6%)	51 (48.1%)	30 (28.3%)	
Left	115 (52.0%)	23 (20.0%)	62 (53.9%)	30 (26.1%)	
Histological type					**0.0001**
SSM	151 (68.3%)	33 (21.9%)	88 (58.3%)*	30 (19.9%)	
NM	43 (19.5%)	14 (32.6%)	21 (48.8%)	8 (18.6%)	
ALM	21 (9.5%)	0 (0%)	0 (0%)	21 (100%)*	
LMM	4 (1.8%)	0 (0%)	3 (75%)	1 (25%)	
Others	2 (0.9%)	1 (50%)	1 (50%)	0 (0%)	
Size (*n* = 218)	12.47 ± 1.69	10.13 ± 4.82	12.62 ± 7.02	13.98 ± 10.02*	**0.036**
Breslow (*n* = 221)					0.061
** < **1 mm	**91 (41.1%)**	**24 (26.4%)**	**50 (54.9%)**	**17 (18.7%)**	
** > **1—2 mm	**56 (23.3%)**	**10 (17.9%)**	**30 (53.6%)**	**16 (28.6%)**	
** > **2—4 mm	**49 (22.1%)**	**11 (22.4%)**	**24 (49.0%)**	**14 (28.6%)**	
** > **4 mm	**25 (11.3%)**	**3 (12.0%)**	**9 (36.0%)**	**13 (52.0%)**	
Ulceration (*n* = 221)	50 (22.6%)	14 (28.0%)	16 (32.0%)	20 (40.0%)*	**0.008**
Regression (*n* = 185)	38 (20.5%)	7 (18.4%)	23 (60.5%)	8 (21.1%)	0.270
TIL (*n* = 187)					0.069
No	42 (22.5%)	11 (26.2%)	13 (31.0%)	18 (42.9%)	
Mild (< 15%)	84 (44.9%)	19 (22.6%)	43 (51.2%)	22 (26.2%)	
Intense (> 50%) Missing: 34	61 (32.6%)	11 (18.0%)	36 (59.0%)	14 (23.0%)	
Mitosis (*n* = 139)	3.72 ± 5.36	2.77 ± 2.74	3.35 ± 4.34	5.04 ± 7.73	0.152
LVPNI (*n* = 162)	24 (14.8%)	1 (4.2%)	7 (29.2%)	16 (66.7%)*	**0.0001**
Satellitosis (*n* = 89)	6 (6.7%)	0 (0%)	2 (33.33%)	4 (66.7%)	0.124
Basins (*n* = 221)	1.00 ± 0.20	0.98 ± 0.14	1.02 ± 0.23	1.00 ± 0.18	0.533
N + (*n* = 214) Missing: 7	40 (18.7%)	8 (20.0%)	18 (45.0%)	14 (35.0%)	0.459
Number of involved nodes after LFN (*n* = 40)	1.57 ± 3.29	0.88 ± 1.80	2.21 ± 4.35	1.13 ± 2.16	0.523

Bold letters are unnecessary as statistical significance is marked with an asterisk in the table 1

So the bold letters should be normal letters in the table 1

*SSM* superficial spreading melanoma, *NM* nodular melanoma, *ALM* acral-lentiginous melanoma, *LMM* lentigo maligna melanoma, *TIL* tumor-infiltrating lymphocytes, *LVPNI* lymphovascular and perineural infiltration, *LFN* lymphadenectomy

Statistical significance is marked with an asterisk

A greater presence of LVPNI was found in lesions located on the foot (66.7% vs 29.2% (leg) and 4.2% (thigh); *p* < 0.0001) and, although it did not reach statistical significance, melanomas of a more distal location had a lower inflammatory cell infiltrate. In fact, a more intense inflammatory infiltrate was seen in middle location lesions (23% (foot) vs 59.0% (leg) vs 18% (thigh); *p* = 0.069).

In relation to the number of lymphatic drainage basins found, only multiple drains (double, specifically found in five cases with independent drains to the inguinal region and popliteal cavity) were found in distal melanomas of the foot and ankle, although there was no significant difference in the average number of lymphatic drainage basins found between the groups (*p* = 0.533).

No differences were found between the groups in terms of sentinel node involvement, nor in the number of lymph nodes affected after lymphadenectomy following a positive sentinel lymph node.

Multivariate analysis showed no significant differences among the variables with significative relevance in the bivariate analysis. Only the anatomical location presented statistical significance in the Cox regression and, therefore, discriminates cases with a higher mortality risk and a lower disease-free survival rate among distal melanomas, mainly the foot (worse prognosis) compared with more proximal melanomas like the thigh (Table 2).

**Table 2:** Cox regression of the variables for melanoma-specific survival (MSS) and disease-free survival (DFS)

MSS	*p*	OR (IC95%)
Histological type	0.885	–
Size	0.606	–
Breslow index	0.551	–
Ulceration	0.075	–
LVPNI	0.865	–
Location		
Foot	Refere nce	
Leg	0.097	–
Thigh	0.032	0.299 (0.09–0.904)

DFS	*p*	OR (IC95%)
Histological type	0.706	–
Size	0.913	–
Breslow index	0.598	–
Ulceration	0.098	–
LVPNI	0.085	–
Location		
Foot	Reference	
Leg	0.082	–
Thigh	0.032	0.410 (0.182–0.927)

*LVPNI* lymphovascular and perineural infiltration

**Fig. 1:**
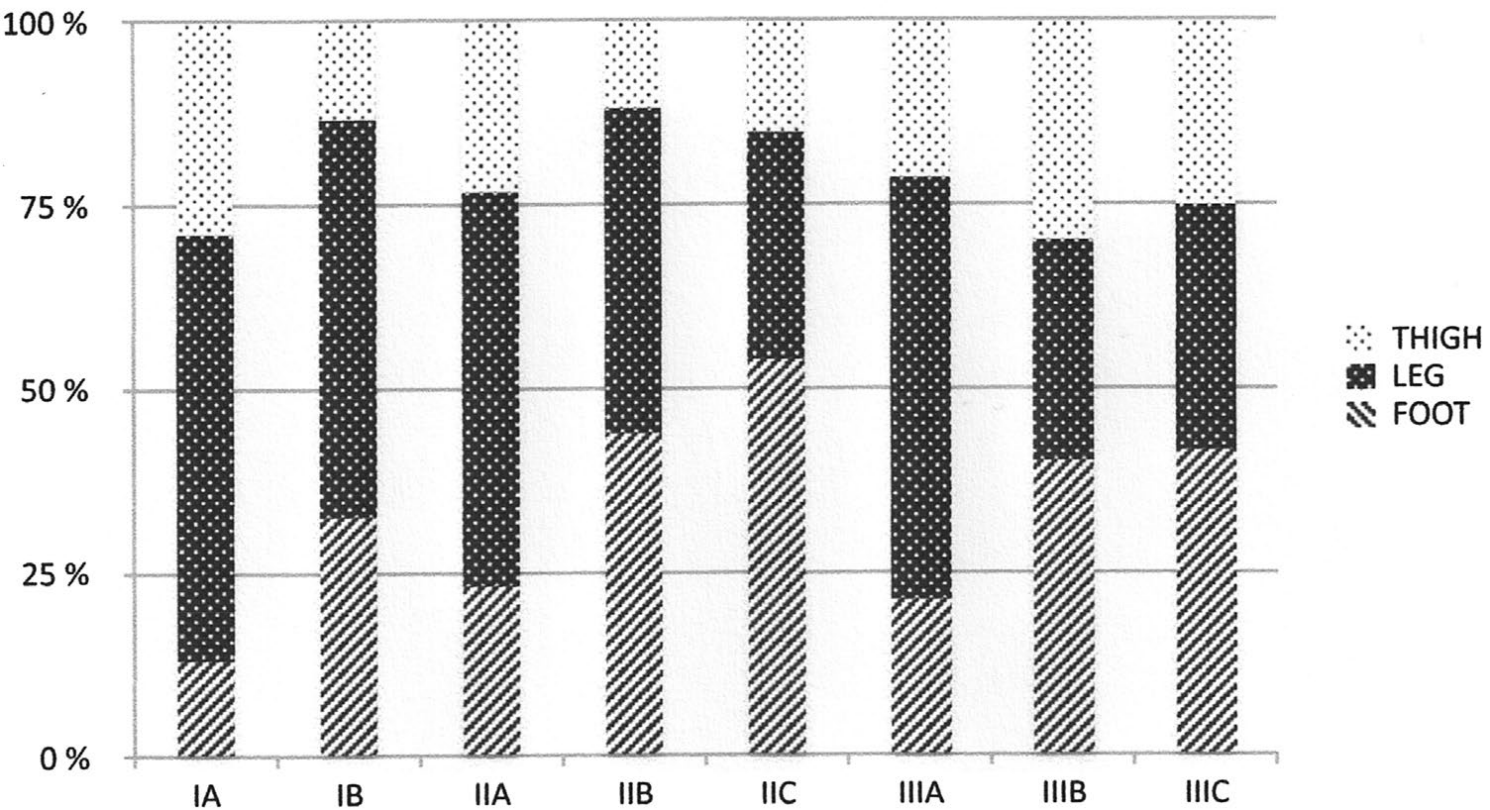
Distribution of the patients from the three localization groups according to the 7th edition of the TNM staging. Stage III disease was established for the final staging following sentinel node biopsy and lymph node dissection when indicated. *p* = 0.163

Figure 1 shows the distribution of the three localization groups according to the 7th edition of the TNM classification. A higher proportion of distal melanomas at more advanced stages was observed but no statistically significant differences were found.

**Fig. 2:**
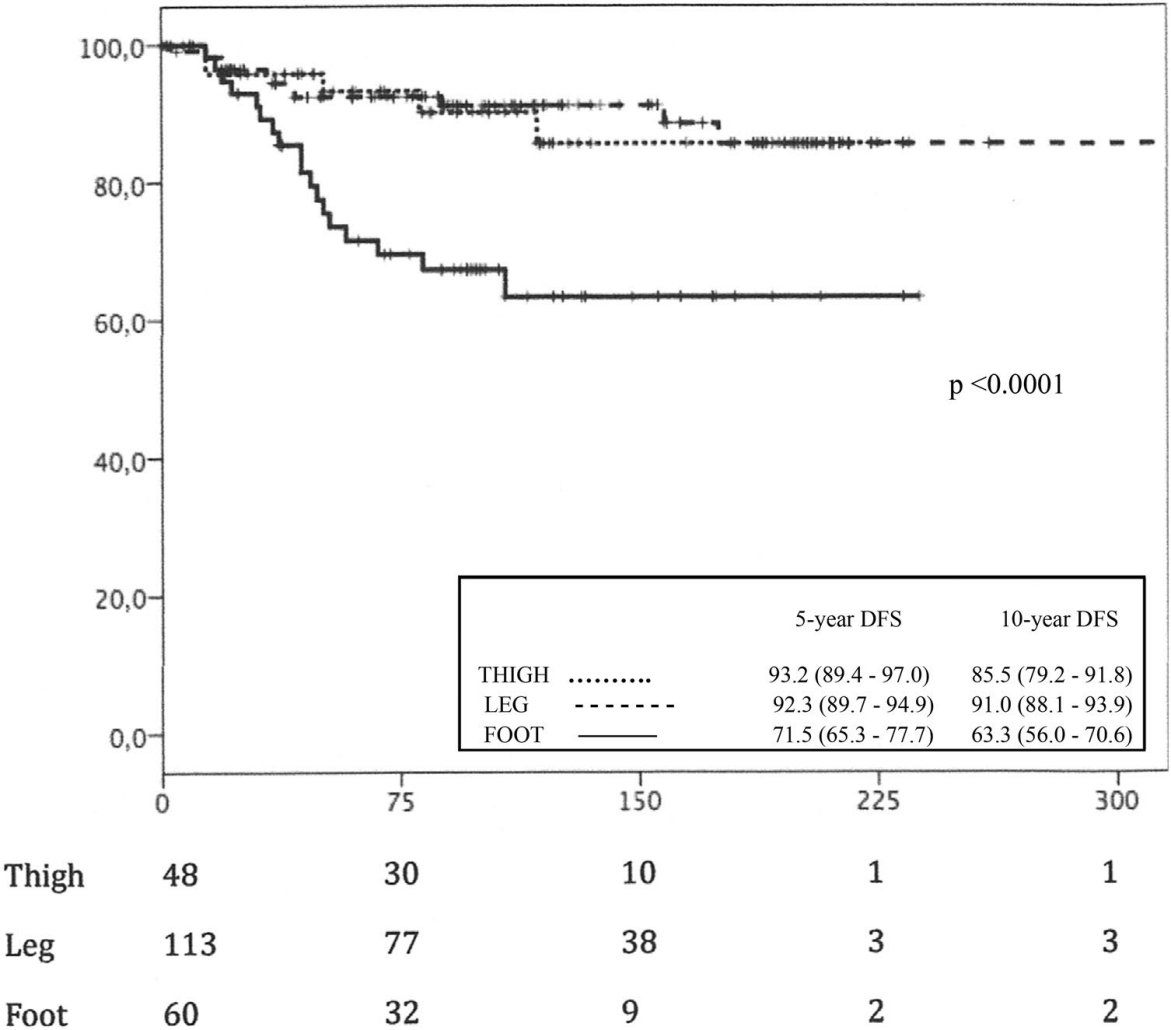
Melanoma-specific disease-free survival (DFS), in months

**Fig. 3:**
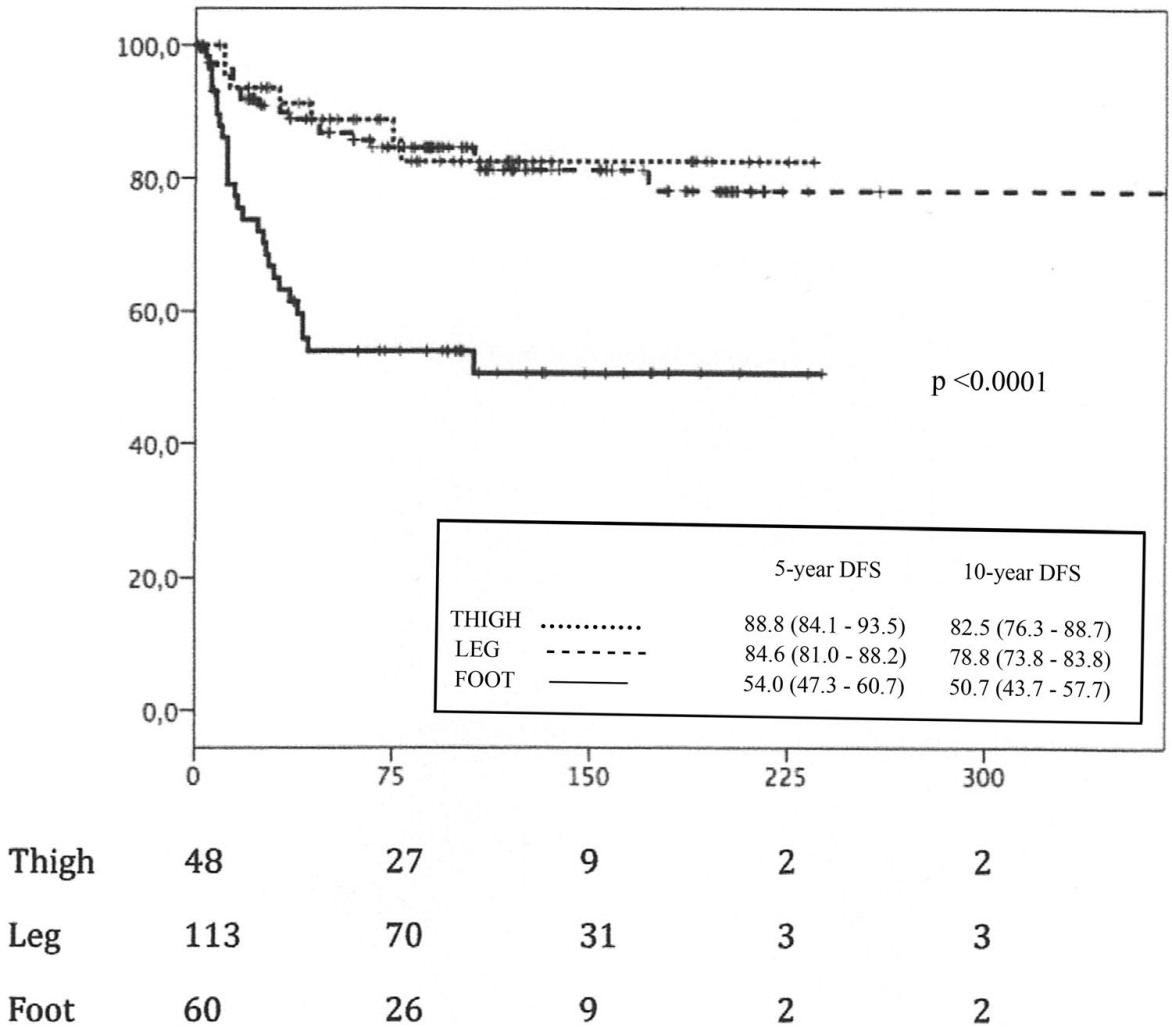
Melanoma-specific survival (MSS) in months

Regarding survival rates, significant differences were found in both the DFS (*p* < 0.001) and the MSS (*p* < 0.001) for the most distal locations on the foot and ankle, which presented a lower melanoma-specific overall survival (168.73 ± 13.32 months) compared to more proximal locations (210.78 ± 10.14 months—thigh—and 327.85 ± 11.99 months—leg—) and a lower melanoma-specific disease-free survival (133.43 ± 14.61 months—foot—) than the more proximal ones (201.22 ± 11.69 months—thigh—and 301.17 ± 14.33 months—leg—). The survival curves for DFS and MSS with survival rates at 5 and 10 years and 95% confidence intervals are shown in Figs. 2 and 3, respectively.

## Discussion

In this study, the characteristics of 221 patients with lower limb cutaneous melanoma, without evident regional or distant metastasis in their initial diagnosis, are analyzed, and survival data are compared according to their location on the thigh, leg or foot.

Regarding the general distribution of lesions, 27.1% are ankle or distal cases, figures that are similar to those seen in recent series with the same definition of distal lesion—foot and ankle—and which range from 19 to 33% [8, 9]. It should be mentioned that plantar melanomas, an important prognostic subgroup of distal melanomas for some authors [7, 11], are more frequent in the Black and Asian population whereas this study only presented Caucasian patients. However, older Caucasian women have been reported as a more frequent group for melanoma of the foot [13].

Bivariate analysis shows that the mean age of patients with foot melanoma is somewhat older than that of those on the leg or the thigh, but with no statistically significant differences found. This information also falls within the age range reported by other authors [8, 9, 14]. Something similar also occurs with the gender distribution: a third of the patients are men and two thirds are women, coinciding with the reportings in other published series [8, 9, 12].

A significant relationship was found between the melanoma being located on the foot or the ankle and a higher Breslow index, confirming the results seen in previous studies [8, 9]. This relationship has also been found with the lesion diameter at the time of diagnosis, a variable that was vaguely analyzed in previous studies and could be explained by the delay in diagnosis. On the other hand, the association of a distal location with other histopathological variables that confer a worse prognosis, such as ulceration and LVPNI, would also support the interpretation of being in the presence of a more advanced lesion due to a delay in the diagnosis. Patients will usually seek medical consultation later in these cases as the lesions are less clinically evident, or they have problems with the initial differential diagnosis, being interpreted as vascular alterations, infectious problems or subungual hematomas, which often lead to them being noticed later on by the patient, sometimes ignored by physicians and often misdiagnosed [15]. These findings coincide with those provided by other series [8, 9] with such delays in suspicion and diagnosis possibly leading to a worse survival rate.

The findings of larger, thicker lesions and a greater presence of ulceration also support the idea that the possible prognostic differences between the most distal lesions compared to the most proximal ones, could be due to a delay in the detection and, therefore, of the melanoma diagnosis [5]. This is confirmed in the data referring to the TNM classification, which presents a higher proportion of more advanced cases among the distal melanomas.

In this context, survival analysis showed significant differences in both MSOS and MSDFS in favor of proximal lesions compared to distal lesions. In fact, this allows us to differentiate those located on the foot or the ankle with a worse prognosis, from the rest of the locations on the lower limb that have a similar prognosis between them. Another aspect used to explain this worse prognosis has been that the different regions of skin present different lymphatic densities, along with the fact that distal lesions have a longer path in the lymphatic drainage system, and theoretically a greater chance of extending [16]. Conversely, the absence of differences observed with lymph node involvement would not support this theory.

The cases studied include plantar and subungual melanomas, typical of the distal group, which could help explain the worse prognosis [11], although there are other studies that, in comparing plantar melanoma with all other types of melanoma, find differences in histopathological prognostic variables but do not show differences in survival rates [6, 17]. In fact, in the publication by Bauert et al. [17], the survival rate for plantar melanoma is mainly related to the lesion thickness and, as in the present series which does not show statistical significance for lesion thickness in the multivariate analysis, the theoretical negative influence of acral melanoma on the prognosis is not observed.

As advocated by other authors the results of the present study show the negative effect on both MSS and DFS for lesions located on the foot, regardless of the absence of factors classically considered as poor prognosis, such as the number of mitosis or acral histology [6]. This shows the importance of carrying out further studies to characterize better the prognosis of certain lesion subtypes included in this study within the group defined as distal, especially the subungual and plantar lesions, and to include an analysis according to the new WHO histological classification [18]. In this context, plantar mechanical stress has been proposed as a production mechanism, with studies showing differences in the appearance of melanomas in weight-bearing regions of the plantar foot and that, perhaps, this could be a factor that encourages tumor dissemination [19–21].

Another possible explanation, in this case of the better prognosis of lesions located on the thigh, would be the difference in the thickness of the skin, which, being greater in this location compared to that of the leg or the foot, would make the superficial vascular plexus remain more than 1 mm deep and, theoretically more difficult for the tumor cells to reach the vessels with the same Breslow index.

Among the limitations of this study, it could be argued that this is a small, multicentre series, although as a prospective series of routine clinical practice, it should be considered representative of the real-life experience of these patients, which could provide a greater utility for the assessment of the results.

In conclusion, the results obtained confirm the hypothesis that the more distal location of lower limb cutaneous melanoma is a relevant prognostic factor, and this fact must be considered to prevent them going unnoticed and therefore delaying diagnosis.
